# Genetics in Idiopathic Pulmonary Fibrosis: A Clinical Perspective

**DOI:** 10.3390/diagnostics12122928

**Published:** 2022-11-23

**Authors:** Spyros A. Papiris, Caroline Kannengiesser, Raphael Borie, Lykourgos Kolilekas, Maria Kallieri, Vasiliki Apollonatou, Ibrahima Ba, Nadia Nathan, Andrew Bush, Matthias Griese, Philippe Dieude, Bruno Crestani, Effrosyni D. Manali

**Affiliations:** 1 2nd Pulmonary Medicine Department, General University Hospital “Attikon”, Medical School, National and Kapodistrian University of Athens, 12462 Athens, Greece; 2 Département de Génétique, APHP Hôpital Bichat, Université de Paris, 75018 Paris, France; 3 INSERM UMR 1152, Université de Paris, 75018 Paris, France; 4 Service de Pneumologie A, INSERM UMR_1152, Centre de Référence des Maladies Pulmonaires Rares, FHU APOLLO, APHP Hôpital Bichat, Sorbonne Université, 75018 Paris, France; 5 7th Pulmonary Department, Athens Chest Hospital “Sotiria”, 11527 Athens, Greece; 6 Peditric Pulmonology Department and Reference Centre for Rare Lung Diseases RespiRare, INSERM UMR_S933 Laboratory of Childhood Genetic Diseases, Armand Trousseau Hospital, Sorbonne University and APHP, 75012 Paris, France; 7 Paediatrics and Paediatric Respirology, Imperial College, Imperial Centre for Paediatrics and Child Health, Royal Brompton Harefield NHS Foundation Trust, London SW3 6NP, UK; 8 Department of Pediatric Pneumology, Dr von Hauner Children’s Hospital, Ludwig-Maximilians-University, German Center for Lung Research, 80337 Munich, Germany; 9 Department of Rheumatology, INSERM U1152, APHP Hôpital Bichat-Claude Bernard, Université de Paris, 75018 Paris, France

**Keywords:** interstitial lung disease, idiopathic pulmonary fibrosis, alveolar epithelial cell, short telomere syndrome, surfactant-related gene mutations, telomere-related-gene mutations, single nucleotide polymorphisms, MUC5B

## Abstract

Background: Unraveling the genetic background in a significant proportion of patients with both sporadic and familial IPF provided new insights into the pathogenic pathways of pulmonary fibrosis. Aim: The aim of the present study is to overview the clinical significance of genetics in IPF. Perspective: It is fascinating to realize the so-far underestimated but dynamically increasing impact that genetics has on aspects related to the pathophysiology, accurate and early diagnosis, and treatment and prevention of this devastating disease. Genetics in IPF have contributed as no other in unchaining the disease from the dogma of a “a sporadic entity of the elderly, limited to the lungs” and allowed all scientists, but mostly clinicians, all over the world to consider its many aspects and “faces” in all age groups, including its co-existence with several extra pulmonary conditions from cutaneous albinism to bone-marrow and liver failure. Conclusion: By providing additional evidence for unsuspected characteristics such as immunodeficiency, impaired mucus, and surfactant and telomere maintenance that very often co-exist through the interaction of common and rare genetic variants in the same patient, genetics have created a generous and pluralistic yet unifying platform that could lead to the understanding of the injurious and pro-fibrotic effects of many seemingly unrelated extrinsic and intrinsic offending factors. The same platform constantly instructs us about our limitations as well as about the heritability, the knowledge and the wisdom that is still missing.

## 1. Introduction

Fibrotic interstitial lung diseases (f-ILDs) are a heterogeneous group of diffuse parenchymal lung disorders that may share common overlapping clinical characteristics and, under not yet clearly identified circumstances, progression to pulmonary fibrosis [1,2,3,4]. Variable susceptibility and progression to diffuse lung fibrosis constitute the major determinant of their unpredictable clinical course and prognosis [5,6,7,8]. A major achievement of the last decades in the elucidation of their etiopathogenetic mechanisms, besides the role of environmental, aging-related, immune or other factors, is that genomics play an important, previously unsuspected, role in their development [9,10,11]. f-ILDs include idiopathic interstitial pneumonias (IIPs), autoimmune-rheumatic-disorder-related lung fibrosis, hypersensitivity pneumonia, sarcoidosis, and unclassified and rare f-ILDs [12,13,14]. Idiopathic pulmonary fibrosis (IPF), the “core” of all f-ILDs, is the most common and clinically severe, bears the histology of usual interstitial pneumonia (UIP), and although it invariably presents an ominous prognosis despite current pharmacological treatment, its clinical course is highly unpredictable, lasting at diagnosis from months to almost a decade [1,15,16]. Most of the progress on genomics in f-ILDs is in IPF, where genetic variants underlie 30% of patients at risk of developing sporadic or familial IPF (two or more cases of IPF in the same family within three degrees of relationship) [17]. The same genetic susceptibility does not lead to identical clinical, imaging, or histological phenotypes in either the sporadic or familial context, delineating the role of epigenetic factors [18,19].

The traditional view that IPF is a sporadic disease of unknown etiology limited to the lungs and almost always affecting the elderly has been challenged in the last decade by the clinical observation that it develops 10 times more often in members of families and that it may develop as part of a multisystem, inherited disease of telomeres, named telomeropathy (short telomeres syndrome, STS) where members of the same family may also present skin, mucosa, and hair abnormalities, T-cell immunodeficiency, and bone-marrow and/or liver disease [20,21,22,23,24,25]. All the above, in combination with the fact that certain forms of interstitial lung disease develop at a very early age (children’s interstitial lung disease-chILD) may be attributed to genetic predisposition, provided evidence of an inheritable component in IPF [26].Thanks to progress in genomics, it has been shown that heritability in adult IPF relates, in most cases but not all, to pathogenic variations in surfactant-related genes (*SRG*s) and telomere-related genes (*TRG*) in monogenic inheritance and a single-nucleotide polymorphism (SNP) in the promoter of the *MUC5B* gene encoding Mucin 5B (rs35705950 T risk allele) in polygenic inheritance (the most common genetic variant) [27]. Genetic studies in IPF and also in other f-ILDs have identified several SNPs associated with fibrosis, the rare variants conferring a higher risk while the common ones a lower risk [28]. Genome-wide-association studies continue to identify new candidate chromosomal loci to be associated with increased susceptibility to IPF [29,30,31]. The discovery in IPF that multiple genetic components, including the rarest variants, may be encountered in the same patient broadens our understanding of clinical heterogeneity [32,33,34], suggesting co-responsibility in the pathogenetic mechanisms of the disease instead of a mere cohabitation and necessitating a unifying pathogenetic theory regarding genetic risk. Differences in genomics in IPF patients may drive differences in the disease course (phenotypic unpredictability) through the diversity of pathogenetic mechanisms and in the near future may lead to a personalized approach towards precision in the cure of a currently incurable disease [32,35,36]. The aim of the study is to provide an overview of genetics in IPF from a clinical perspective.

## 2. Overview of IPF Pathogenesis

Despite considerable progress and decades of research unraveling the complexity of mechanisms underlying the pathophysiology of IPF, a unifying and credible hypothesis linking the multiplicity of cell types, molecular factors, and signaling pathways in its pathogenesis still remains elusive [37,38,39]. Understanding its pathology, in essence the process of lung wound healing, appears clearly the first step in the elucidation of IPF pathogenesis [40,41,42,43,44]. Parenchymal lung fibrosis, the end of the pathogenetic process, begins or appears early on in the subpleural basal and posterior peripheral areas of the lungs, sites of increased traction forces, and is characterized by extreme remodeling of the alveolar spaces and walls including the distal bronchioles (traction bronchiolectasis-bronchiectasis) [45,46,47]. The histopathological hallmarks include the bronchiolization of the distal airspaces, honeycombing, fibroblastic foci, and abnormal epithelial hyperplasia defining the UIP histology [48]. Fibrosis constitutes of an aberrant non-resolving extracellular matrix deposition and necessitates multiple cells, fibrogenic molecules, and signaling pathways triggered by several asymmetrical injuring factors, acting repeatedly on alveolar epithelial cells (AEC), according to the current pathogenetic theories [49]. Injured AECs due to exposure to inhaled adverse factors acting on a susceptible individu-al background due to ageing and susceptible genome leads to senescence of AECs and through a multitude of pathways acting on fibroblasts and myofibroblasts alters extracel-lular matrix leading to aberrant and self-perpetuating wound healing [50,51,52]. Indeed, it is known that AEC2s are progenitor cells for AEC1s (which cover most part of the alveolar surface) in a normal lung. After damage, either acute or subacute, of the alveolar epithelium, AEC2s proliferate and transdifferentiate into AEC1s. Senescent and dysfunctional AEC2s are not able to transdifferentiate into AEC1s, but activate differential pathways, e.g., those involved in epithelial–mesenchymal transition that eventually lead to aberrant repair (ECM deposition) without regeneration and to fibrosis. [53]. Genetic predisposing factors may stand alone or even coexist with most of the above predisposing conditions and through a diversity of pathogenetic mechanisms may lead to a “homogeneous” pattern of fibrotic lung disease, determine its earlier appearance, influence clinical course and prognosis, and possibly in the future direct early therapeutic measures [29,54,55,56,57]. The identification of high-risk populations may also guide early referral and the adoption of preventive measures that could postpone the development of the disease [58,59,60,61,62,63,64]. However, in familial PF, despite considerable progress, the identification of predisposing genetic variants still eludes us in a significant proportion of patients [65]. 

## 3. Historical Perspective on Genetics in IPF

**Figure 1:**
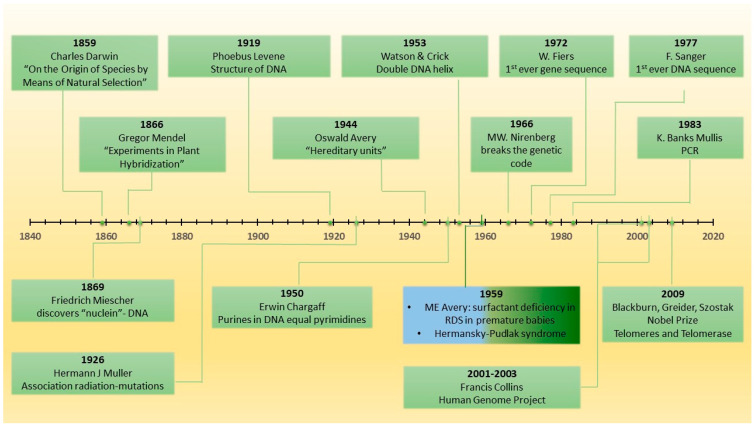
Major discoveries in the history of genetics: 1859: Ch. Darwin: “On the Origin of Species”; 1866: Gr. Mendel: Experiments in Plant Hybridization”; 1869: Fr. Miescher: “nuclein” (DNA); 1919: Ph. Levene: DNA structure; 1926: HJ Müller: radiation and lethal mutations; 1944: O. Avery, C. MacLeod, M. McCarty inheritance through “hereditary units”; 1950: E. Chargaff: “the total number of purines in DNA is equal to the total number of pyrimidines”; 1950s: JA Clements: physical properties of surfactant; 1953: J. Watson and F. Crick: three-dimensional double helical structure of DNA molecule; 1959: ME Avery: surfactant deficiency in RDS in premature babies; 1959: Hermansky–Pudlak syndrome; 1966: MW. Nirenberg break the genetic code; 1972: W. Fiers: first sequence of a gene; 1977: F. Sanger: sequence DNA for the first time; 1983: K. Banks Mullis: polymerase chain reaction; 2001–2003: Francis Collins: Human Genome Project; 2009: EH. Blackburn, CW. Greider, JW. Szostak: Nobel Prize “for the discovery of how chromosomes are protected by telomeres and the enzyme telomerase”.

**Figure 2:**
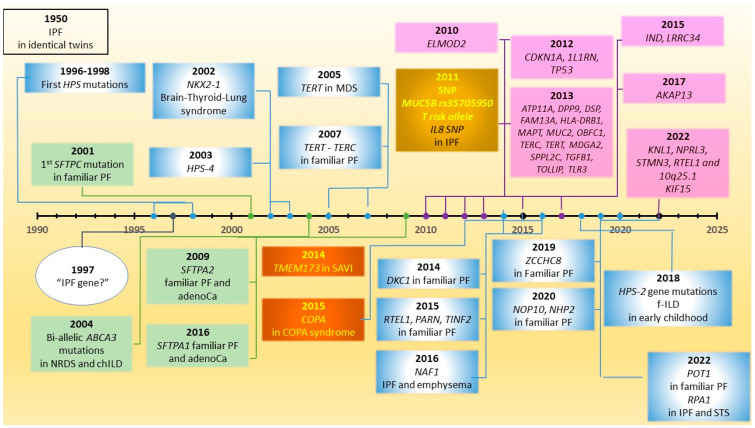
Discoveries in IPF genetics: 1950: IPF is reported in identical twin sisters; 1996–1998: First mutations in Hermansky–Pudlak syndrome (*HPS)* genes; 1997: Is there is fibrosis gene in pulmonary fibrosis? 2001: First *SFTPC* mutation in familiar IP; 2002: *NKX2-1* mutations in brain-thyroid-lung syndrome; 2003: *HPS-4* mutations; 2004: Bi-allelic *ABCA3* mutations in NRD syndrome and chILD; 2005: Mutations in *TERT* in patients with bone-marrow failure; 2007: *TERT* and *TERC* mutations in familiar IP; 2009: *SFTPA2* mutations FIP and adenocarcinoma families, 2010: *ELMOD2* SNP polymorphism in IPF; 2011: A common SNP in the promoter region of the gene *MUC5B*: the strongest polygenic risk factor for sporadic IPF and familiar IP; 2011: *IL8* SNP polymorphism in IPF; 2012: *CDKN1A, 1L1RN, TP53* SNP polymorphisms in IPF; 2013: *ATP11A, DPP9, DSP, FAM13A, HLA-DRB1, MAPT, MUC2, OBFC1, TERC, TERT, MDGA2, SPPL2C, TGFB1, TOLLIP, TLR3* SNP polymorphisms in IPF; 2014: *DKC1* mutation in familiar IP; 2014: *TMEM173* mutations in SAVI, 2015: *RTEL1*, *PARN* and *TINF2* mutations in familiar IP; 2015: *COPA* mutations in COPA syndrome; 2015: *IND, LRRC34* SNP polymorphisms in IPF; 2016 *SFTPA1* mutations in FIP and/or adenocarcinoma families; 2016: *NAF1* mutations in pulmonary fibrosis-emphysema; 2017: *AKAP13* SNP polymorphism in IPF; 2018: *HPS-2* gene mutations: fibrosing lung disease early in childhood; 2019: *ZCCHC8* mutations in FIP; 2020: *NOP10* and *NHP2* mutations in FIP; 2022: *POT1* mutation in FIP; 2022: *KNL1, NPRL3, STMN3, RTEL1*, and an intergenic variant in 10q25.1 in IPF; 2022: *KIF15* mutations in IPF; 2022: *RPA1* mutations in pulmonary fibrosis and STS.

Genetics have revolutionized the way we approach human disease and IPF is not an exception to this rule (Figure 1) [66,67,68]. Inheritable PF has been the focus of intense research since 1950, when the first cases of familial PF in identical twins were reported, raising questions about the presence of a “fibrosis gene” (Figure 2) [69,70]. 

## 4. Surfactant-Protein-Related Genes

In 2001, a breakthrough occurred when a mutation in the gene encoding surfactant protein C (SFTPC) in a young mother and a child with familial ILD was identified [71]. In 2002, the *SFTPC* mutation was identified in a large family, including adults with UIP and children with nonspecific ILD [72]. Thereafter, many studies confirmed that *SFTPC* mutations may cause familial lung disease in an autosomal dominant pattern with very high penetrance in all age groups, from the newborns to the elderly [73,74]. In 2009 and 2016, it was shown that very rare mutations in the genes encoding the surfactant proteins A2 and A1—respectively, SFTPA2 and SFTPA1—segregated with an ILD and adenocarcinoma phenotype in large families with multiple members with ILD occurring at a wide age range and/or adenocarcinoma only in adults [75,76]. The spectrum of *SRGs* implicated in inheritable PF was further expanded by the observation that bi-allelic mutations in the gene of ATP-binding cassette subfamily A, member 3 (ABCA3) protein, which plays a cardinal role in surfactant homeostasis, are associated with neonatal respiratory distress syndrome (RDS) and in “chILD” surviving into adulthood [77,78,79,80]. Mutations in NKX2-1, which encodes thyroid transcription factor-1, regulating the transcription of surfactant proteins and ABCA3, as well as many other lung proteins, was found to be associated with ILD in the context of “brain-thyroid-lung” syndrome (neurological symptoms, peripheral hypothyroidism, and f-ILD). Lung involvement ranges from neonatal RDS to adult PF inherited through an autosomal dominant pattern with variable penetrance. In 25% of patients, it develops as an isolated phenotype and in another 19% in association only with neurological symptoms [81,82,83,84]. Disease-causing mutations in the gene encoding surfactant protein D (*SFTPD*) have not yet been identified, whereas bi-allelic mutations in the gene encoding surfactant protein B (*SFTPB*) are typically associated with neonatal RDS; no adults have been reported so far [85,86]. *SRG* pathogenic variations seem to be implicated in the development of pulmonary fibrosis by disrupting AEC2 homeostasis. Very recently it has been shown that mutations in *SFTPC* lead to accumulation of the mutated protein at the plasma membrane through abnormal recycling from endosomes and from impaired internalization into multivesicular bodies with still unknown effects on the signaling within the AEC2 or its neighbors and the surface expression of otherwise unrelated proteins. Mutations in *SFTPA2* and *SFTPA1* are thought to abolish the secretion of mutant proteins, leading to their sequestration in the AEC2. Conversely, mutations in *ABCA3* and *NKX2-1* result in dysfunctional lamellar bodies and disrupt surfactant homeostasis in AEC2s, which play the cardinal role in alveolar maintenance and repair of recurrent epithelial cell injury of any etiology [87,88,89,90,91,92,93,94,95,96,97,98,99]. 

## 5. Clinical Implications of Carriership of *SRG* Mutations 

Depending on the cohorts, the contribution of *SRG* mutations in monogenic pulmonary fibrosis ranges from 2 to 5%, with the exception of some populations where it may reach 25% in chILD [10,26,74,100,101,102,103,104]. Patients who are carriers of *SRGs* present a great heterogeneity of phenotypes, from lethal neonatal RDS to, more rarely, chILD and adult ILD [74]. In adults, a higher degree of suspicion is required, especially in early-onset disease and in familial PF, where lung fibrosis and/or pulmonary adenocarcinoma segregate in many family members. Gender and environmental exposures do not seem to play a significant role in the development of the disease. In adult patients, the age at which *SRGs*-ILD has been reported ranges from 19 to 71 years and depends on the underlying mutation. The median age for *SFTPC* is 37 years, whereas for *SFTPA1/SFTPA2* is 48 years [99]. *ABCA3* mutations-related ILDs have been described in cases of chILD surviving into adulthood and in adults [79,80,105,106,107]. The radiological imaging patterns conform more often to unclassifiable ILD with reticular abnormalities, ground glass opacities and scattered cystic lesions and may lead to the decision for lung biopsy, often disclosing a UIP pattern and in some association with non-specific or desquamative interstitial pneumonitis pattern or even pulmonary alveolar proteinosis (PAP)-like histology [108,109,110]. The major clinical implication so far described in *SRG* mutations relates to the high frequency (37%) of lung carcinoma (mostly of the adenocarcinoma type) alone (12%) or in combination with pulmonary fibrosis (25%) in *SFTPA1/SFTPA2* adult carriers. Therefore patients should be regularly screened, especially after the age of 40 years [74,76,99] and family members genetically tested for the presence of *SFTPA1/SFTPA2* mutations. No effective treatment exists for surfactant-related genetic disease and there is no consensus on it; studies report treatment with azithromycin or hydroxychloroquine, while others opt for the immunosuppressants azathioprine, methylprednisolone, and cyclophosphamide with no curative or long-term stabilizing effects [111,112]. Gene-based therapies as well as functional rescue therapies of misfolding *ABCA3* mutations by molecular correctors are promising for the future [113,114]. For the moment, bilateral lung transplantation remains the best option for end-stage fibrotic chILD and adult ILD related to *SRG* mutations [74,115].

## 6. Telomere-Related Genes and Telomeropathy

In 2007, two independent research groups reported the association of mono-allelic telomerase mutations and the development of familial PF in adults [116,117]. Their observations initially regarded only mutations in telomerase reverse transcriptase (*TERT*) and the RNA component of the telomerase complex (*TERC*), both responsible for telomere length maintenance, and were partly inspired by the study of the genetic background of the inheritable multisystem disease dyskeratosis congenita, where mutations in the telomerase pathway were initially recognized to cause STS characterized by bone-marrow and liver failure and fibrotic ILD at a young age [118]. Dyskeratosis congenita (DC), was the first described telomeropathy; it consists of an inherited bone-marrow failure syndrome clinically characterized by a muco-cutaneous triad (skin pigmentation, nail dystrophy, mucosal leukoplakia), bone-marrow failure, immune deficiency, pulmonary fibrosis, liver cirrhosis, and the insurgence of malignancies. The discovery in the last 15 years that telomeropathy is a disease related to defects in telomere maintenance with short telomeres and mutations affecting telomerase activity, assembly, and telomere integrity has enabled scientists to consider the telomerase-complex pathway in the pathogenesis of familial PF [23,119,120,121,122,123,124,125]. The genes implicated in telomeropathy are: *ACD, CTC1,DCLRE1B, DKC1, NHP2, NOP10, PARN, POT1, RPA1, RTEL1, STN1, TERC, TERT, TINF2, WRAP53*, and *ZCCHC8.* Mono-allelic or bi-allelic pathogenic variants of a few genes are identified in patients with different clinical spectrum. Severe forms of DC and Höyeraal–Hreidarsson, Revesz, or Coats plus syndromes are more often associated with biallelic variants, while heterozygous *TERT, TERC, RTEL1, PARN, ACD, NHP2*, and *NOP10* variants are found in PF patients(Table 1) [126]. Thereafter, the discovery of mutations in numerous genes of the telomere-maintenance pathway established the role of this pathway in the development of accelerated aging and monogenic PF and led to the acknowledgement of *TRG* pathogenic mutations as the major monogenic cause of IPF, the genetic basis of 30% of cases of familial PF [127,128,129,130,131,132,133,134,135,136,137,138,139]. The net effect of telomere-shortening on somatic cells beyond a critical point is DNA damage leading to apoptosis in high-turnover tissue cells and senescence in low-turnover cells such AECs, and loss of their capacity for resilience and repair leading to repeated injury due to internal or external hits and to lung parenchyma pro-fibrotic remodeling [140]. The effect of *TRG* germline mutations on telomere length is systemic, affecting not only the lung but also the bone marrow and liver. More than 70% of patients that carry *TRG* mutations develop monogenic PF, considered to be the most prevalent “telomeropathy” manifestation. Extra-pulmonary signs or diseases could also be present with variable penetrance: early hair greying, bone-marrow failure syndrome, immunodeficiency-related risk of infections, and liver disease. Interestingly, 15% of patients with familial PF are found to have short telomeres and develop extrapulmonary manifestations (personal or familial) of STS without any identifiable *TRG* mutation [141,142,143]. Short telomeres in familial PF patients and their families may represent a surrogate for *TRG* pathogenic mutations [144,145]. Shortened telomeres are also observed in a significant proportion of sporadic IPF cases, but in that case *TRG* pathogenetic mutations are disclosed in less than 2% of adult patients [146,147,148,149,150,151,152,153]. *TRG*-mutation-associated ILDs are very rare in pediatric patients outside the context of syndromes such as DC, but are increasingly recognized [154].

## 7. Clinical Implications of Carriership of Pathogenic Variations in Telomere-Related Genes

Approximately 30% of patients with familial PF are carriers of pathogenic variations in *TRGs*. Elaborate genealogical research studies have shown that a latency period of >300 years may pass before the cumulative effect of telomere shortening eventually leads to familial PF in *TRG* mutation carriers [141,155]. In addition to personal/familial extra-pulmonary abnormalities suggestive of STS involving the skin, the liver, and the bone marrow, the disease is uniformly progressive, irrespective of its phenotypic heterogeneity, and bears a reduced transplant-free survival time [18,19,32,156,157]. The increased risk for complications and high mortality post-lung-transplantation is due mostly to sepsis, greater than expected bone-marrow suppression and increased rates of chronic allograft dysfunction and airway complications (dehiscence and stenosis) [156,157,158,159,160]. Subclinical bone marrow and liver abnormalities, including significant T-cell immunodeficiency silently existing in these patients, may aggravate post-lung transplantation-immunosuppression-drug toxicities [161]. Therefore, telomeropathy is now recognized in addition to age, frailty, pulmonary hypertension, cardiovascular risk, and lung cancer as a parameter for which careful consideration must be given to pre-operative optimization, surgical technique, pulmonary rehabilitation, and a tailored immunosuppressive treatment protocol to produce the best post-transplantation outcomes [127,143,162,163,164,165,166]. Bone-marrow failure presenting as myelodysplastic syndrome (MDS) or acute myeloid leukemia (AML) may arise at any timepoint in the disease’s course in almost 10% of patients, especially those younger than 65 years old [18,19,32,141,167,168,169,170]. Naturally occurring somatic mutations in telomere maintenance genes observed in some patients with familial-PF potentially might rescue premature age-related clonal hematopoiesis and transformation to MDS/AML [139,171]. Solid tumors are rare; their risk appears higher in male *DKC1* mutation carriers [141]. Subclinical forms of liver involvement and cryptogenic liver cirrhosis may also affect patients with or without concurrent pulmonary fibrosis and/or bone marrow failure [172,173,174]. Occasionally, liver disease may present as severe unresponsive hypoxemia (hepatopulmonary syndrome) [161,175,176]. Taking a careful clinical and family history focused on extrapulmonary manifestations of STS can provide important prognostic information in patients with IPF, as this is associated with shorter survival [177]. Patients who are carriers of *TRG* mutations should be systematically examined for both pulmonary and extrapulmonary disease severity and progression. It is recommended to stop or limit environmental toxic factor exposure, including tobacco smoke, alcohol, and professional exposures.

Patients who are carriers of *TRG* mutations have so far been treated following the recommendations for patients with sporadic IPF: “a conditional YES for antifibrotics” [33,178,179,180]. Androgens, including danazol, are under investigation [181,182]. The possibility of telomerase gene therapies, the potential for CRISPR editing to lengthen telomeres, or other approaches to target molecular defects associated with aging and *TRG* mutations have been examined but are so far restricted by great obstacles and challenges that probably only a deeper understanding of fundamental lung biology could overcome in the future [127,183].

## 8. Interferonopathies 

### 8.1. STING-Associated Vasculopathy with Onset in Infancy 

(SAVI) is an auto-inflammatory monogenic disease related to heterozygous gain-of-function mutations in STING1, an encoding stimulator of interferon genes (STING). Patients present early in life with features of systemic and peripheral vascular inflammation, vascular and tissue damage (nail dystrophy and gangrene of fingers or toes, nasal septum perforation), low-titer autoantibodies, and f-ILD (Table 1). Pathobiologically, the stimulation of STING activates endothelial cells and induces upregulation of interferon-response genes, apoptosis-pathway genes, and endothelial cell death [184,185,186,187]. 

Aberrant activation of the STING pathway is also detected in COPA syndrome, characterized by dysregulation of the innate and adaptive immune response. Clinically, symptoms occur with varying severity, including ILD with or without pulmonary hemorrhage, inflammatory arthritis, or immune-mediated kidney disease; autoantibodies are evident in most young patients [188]. COPA partially overlaps with SAVI in features such as severe systemic inflammation, recurrent fevers, ILD, early onset in life, and a predominant constitutive type I IFN gene activation. It has been shown that dominant autosomal loss-of-function mutations in the *COPA* gene, which encodes the α-subunit (COPα, COPA) of the coatomer complex I (COPI) lead in COPA deficiency and cause disease through defective retrograde trafficking, specifically the lost Golgi-to-ER retrieval of immune signaling protein STING pathway (Table 1) [189,190,191,192]. An increased specific “interferon signature” associating IFNa dosage and expression of IFN stimulated genes (ISGs) could be found in blood samples of patients with *STING1* or *COPA* mutations. 

### 8.2. Hermansky–Pudlak Syndrome 

Hermansky–Pudlak syndrome (HPS), first described in 1959, is an inherited multisystem autosomal recessive disease characterized by oculocutaneous albinism, bleeding diathesis, inflammatory bowel disease, and pulmonary fibrosis [193,194]. There are 10 genes implicated in HPS: *AP3B1, HPS1, HPS3, HPS4, HPS5, HPS6*, and less commonly, *AP3D1, BLOC1S3, BLOC1S6*, and DTNBP1, inherited in an autosomal recessive manner. Pulmonary fibrosis has so far been described in *HPS1, HPS4,* and *AP3B1* genes (Table 1) [195,196,197,198,199]. Hermansky–Pudlak syndrome type 2 manifests with fibrosing lung disease early in childhood [200]. The pathobiology of the syndrome is related to the defective formation of lysosome-related organelles and abnormal intracellular vesicle trafficking due to mutations in genes regulating protein complexes such as BLOC-1, BLOC-2, BLOC-3, and the AP-3 complex, manifesting in various organs and cells including AEC2; endoplasmic reticulum stress, impaired autophagy, defective surfactant secretion, and altered phospholipid content in association with the dysregulation of alveolar macrophages, triggering the development of lung fibrosis [201,202,203,204,205,206,207,208].

## 9. Clinical Implications of Other Rare Monogenic Forms of Pulmonary Fibrosis

Mutations in genes related to rare syndromic forms of IPF with characteristic extrapulmonary manifestations have historically contributed to the understanding of its heritability and may add to the search for its pathogenesis, unraveling specific pathobiologic pathways [164,209]. The major clinical implications of the genetic analysis in case of extrapulmonary findings consistent with other syndromic forms of pulmonary fibrosis, such as Hermansky–Pudlak, STING, and COPA syndromes, consist mainly in the documentation of the diagnosis and the initiation of specific treatments. The clinical utility of genetic analysis in that case is associated with personalized treatment approaches as well as multidisciplinary collaboration for management by expert centers. The participation in international registries and multicenter clinical trials is the only way that further expert knowledge could be gained for these rare and very challenging diseases [210,211,212,213,214].

## 10. Common Variants in IPF and *MUC5B* rs35705950 

The development of advanced genetic techniques such as genome wide association studies (GWAS) allows the examination of the association of hundreds of variants and the risk of developing IPF. So far, more than 20 mutations in genes implicated in host defense, cell–cell adhesion, and DNA repair have been shown to genetically predispose to the disease, demonstrating that IPF could be considered not only a monogenic but also a polygenic disease with a lot of yet unidentified variants implicated in the susceptibility of the disease and that genetic susceptibility regards not only familial but also sporadic cases [29,30,215]. The common single nucleotide polymorphism (SNP) in the promoter region of the gene *MUC5B* that encodes muc5B protein (MUC5B rs 35705950T allele) as well as the rest of the common SNP associated with IPF is shown in Figure 2 [30,55,216,217,218,219,220,221,222].

In 2011, a common SNP in the promoter region of the gene *MUC5B* that encodes muc5B protein (MUC5B rs 35705950T allele) was reported as the strongest polygenic risk factor for the development of familial PF and IPF [223]. *MUC5B* rs35705950 T risk allele carriership increases the odds ratio in heterozygous and homozygous patients by 6.8 and 20.8 for familial ILD and 9.0 and 21.8 for IPF, respectively [223]. This association was found by several independent groups of researchers initially in IPF patients and later on in other forms of progressive fibrotic lung disease, such as rheumatoid arthritis-UIP-ILD, fibrotic hypersensitivity pneumonitis, asbestosis, and interstitial lung abnormalities (ILAs) [224,225,226,227,228,229,230,231,232]. In this context, pulmonary fibrosis mechanisms relate to the altered biology of the bronchial secretory cell. *MUC5B rs35705950* T risk allele carriership increases the expression of mucin5B in the distal lung, causes mucociliary dysfunction and ER stress, and triggers a vicious cycle of injury/repair and regeneration at the bronchoalveolar junction leading to fibrosis [233,234,235,236,237,238,239,240,241].

## 11. Clinical Implications of Single Nucleotide Polymorphism and *MUC5B* rs35705950 T Risk Allele Carriership

Genome-wide association studies are constantly revealing common SNPs associated with an increased risk for IPF that could be considered in a polygenic transmission model of IPF in contrast to the monogenic one so far described. The clinical implications of those SNPs are not extensively studied with the exception of the *MUC5B* rs35705950 T risk allele and the SNP in *TOLLIP* [242]. *MUC5B* rs35705950 T risk allele carriership is related in both sporadic and familial PF with confident UIP patterns both on HRCT and on histology [243,244,245,246]. In asymptomatic relatives of patients with familial PF as well as in any other individual incidentally found to have ILAs, carriership of *MUC5B* rs35705950 T risk allele is significantly associated not only to the development but also to the progression of PF [230,231,247,248]. In patients with rheumatoid arthritis, *MUC5B* rs35705950 T risk allele carriership has recently been shown to have a significant contribution to the prediction of subclinical RA-ILD disease [249]. Little evidence exists so far that SNPs in common variants could be used to stratify IPF patients and predict response to treatment [250]. Post-hoc analysis in the PANTHER-IPF trial showed that rs3750920 (TOLLIP) TT genotype affected positively the response to N-acetylcysteine (NAC) providing evidence that in a precision-medicine era, genotype-stratified prospective clinical trials should be conducted before any recommendation for therapeutic options in IPF [251]. Despite reports identifying common SNPs and rare missense variants associated with ILD, there are no large multicenter studies that assess the clinical value of screening for rare ILD-linked genetic variants in patients with sporadic IPF.

## 12. Special Considerations 

### 12.1. Children Diagnosed with ILD Reaching Adult Life 

Special consideration is due to genetics in children’s interstitial lung disease (chILD). The range of diseases in that case becomes even broader, including, in addition to *SRG*- and *TRG*-related diseases, many rare and ultra-rare entities, such as surfactant protein catabolic disorders related to *CSFRA*, *CSFRB*, and *OAS1* mutations; syndromic forms of ILD due to intergrin mutations; and chILD related to filamin A variants [25,252,253,254,255]. The scope of the present review could not embrace such a broad spectrum and focuses only on adult disease. However, as the quality of care ameliorates for chILD, survival into adulthood allows a number of those patients to transfer from pediatric to adult care and engages adult pulmonologists to meet their needs [256]. A recent survey demonstrated that the availability of data regarding the characteristics of these patients as well as their transitional care are scarce and tailored the way for immediate action through a dedicated ERS task force whose results are highly anticipated in the near future [257,258].

### 12.2. Genetic Testing for f-ILDs in Everyday Clinical Practice 

At an international level, genetic testing for f-ILDS is not clinically available in every country, nor could it be available in each institution given the increased costs and specialized knowledge that are required. Even more importantly, the interpretation of the results of genetic analysis, especially the pathogenic role of every variant that is identified is a very demanding, time-consuming process that only expert centers can perform. Even in that case, multidisciplinary discussion and international collaboration are often needed. Genetic testing is complete only when genetic counselling is offered to the patients and their families in order to assimilate the clinical, psychological, social, and familial implications of genetic predisposition to disease and adapt accordingly. Based on expert opinion, the clinical indications for which genetic testing may be considered with a high probability of identifying a pathogenic mutation include patients with familial PF, patients with personal or family features of telomeropathy, syndromic forms of the disease, early-onset pulmonary fibrosis without any other obvious explanation, and asymptomatic relatives of patients with known pathogenic disease-causing variants. Genetic testing could also be considered in families where pulmonary fibrosis and adenocarcinoma of the lung segregate in many family members across generations and in patients with ILAs, as well as before lung transplantation. Genetic testing for sporadic PF is not currently recommended [64,65,259].

Official guidelines are still missing; however, expert opinion regarding the examination of relatives of IPF-associated pathogenic variants suggests an initial evaluation at a specialized multidisciplinary outpatient clinic and systematic evaluation thereafter, depending on the individual findings in each case. Genetic analysis may be performed only at the request of a relative who is examined after written informed consent. Depending on the country in question’s legislation, only adult asymptomatic relatives may benefit from a genetic testing. An action plan for the avoidance of toxic exposures is proposed and special care is undertaken depending on the presence or absence of a specific disease. If the genetic analysis confirms the presence of a mutation in an asymptomatic and healthy relative, a regular follow-up is proposed every 3–5 years, unless symptoms suggestive of progressive disease develop earlier [64]. The above recommendations are based on the findings of elaborate cohort studies including first-degree asymptomatic relatives of patients with familial f-ILD that have demonstrated the presence of substantial (25%) subclinical disease [63,260,261,262,263,264]. Early diagnosis of any progressive f-ILD in the familial context could lead to earlier treatment, but whether early treatment translates to improved outcomes is currently unknown and needs to be further explored [265].

## 13. Conclusions—Future Perspective

In the future, the integration of genetic evaluation in the diagnostic algorithm of IPF next to long-standing traditional modalities such as HRCT, BAL, and lung biopsy could support precision medicine and guide the multidisciplinary discussion team to personalized diagnostic and therapeutic approaches. The detection and referral of at-risk populations, including asymptomatic relatives of carriers of known mutations for early recognition of the disease, could optimize clinical outcomes in a currently lately documented and incurable disease. Identifying interstitial lung disease with a genetic background in pediatric populations that survive into adult life could have a tremendous impact on timely diagnosis ensuring a better quality of life, targeted therapies and a successful transition to expert adult care. Genetic counseling regarding the mode of inheritance and the risk to offspring and the other family members provided to all IPF patients with a genetic risk should help informed decision making. Pre-natal and pre-implantation genetic testing bear significant ethical implications and genotype identification and family history are often insufficient to predict the course of disease in an individual and therefore should be handled with caution. 

In the future, the decoding of missing heritability in IPF could further advance our understanding of the disease and offer the opportunity to delay its progression and increase survival. The collaborative work and solidarity of scientific experts and clinicians all over the world regarding the role of genetics in IPF patients is the way to safeguard the tremendous knowledge that genetic science offers, transforming it into healing wisdom.

## Figures and Tables

**Table 1:** Syndromic forms of pulmonary fibrosis.

Telomeropathy Dyskeratosis Congenita	Hermansky–Pudlak	SAVI	COPA
Clinical and Laboratory Manifestations			
Skin: hyperpigmentation, nail dystrophy, hyperhidrosis, hair loss	Oculocutaneous albinism, photophobia, strabismus, nystagmus	Non-healing ulcers on cheeks, nose, fingers, toes, soles	Diffuse alveolar hemorrhage Lung fibrosis
Oral: Leukoplakia, ulcerations, atrophic glossitis, periodontitis, dental carries, lichenoid lesions, hyperpigmentation, neoplasms	Bleeding disorders	Failure to thrive in children	Renal disease lupus nephritis
Lung fibrosis Liver cirrhosis	Lung fibrosis (*HPS1, HPS4,* and *AP3B1* genes)	Lung fibrosis	Arthritis
Bone-marrow failure, anemia	Kidney’s disease	Anemia, leukopenia, thrombocytosis, T-cell lymphopenia	Cystic lung disease
Skull and CNS: microcephalia, cerebral hypoplasia, mental retardation, deafness	Granulomatous colitis	Myositis	
Hypogonadism		Joint stiffness	
Esophageal stenosis			
Osteoporosis			
Short stature			
Skeletal malformations			
Epiphora			
**Genetic Variants**			
		**Interferonopathies**	
17genes implicated: *ACD, CTC1, DCLRE1B, DKC1, NHP2, NOP10, PARN, POT1, RPA1, RTEL1, STN1, TERC, TERT, TINF2, WRAP53*, and *ZCCHC8*	10 genes implicated: *AP3B1, HPS1, HPS3, HPS4, HPS5, HPS6*, and less commonly, *AP3D1, BLOC1S3, BLOC1S6*, and *DTNBP1* inherited in an autosomal recessive manner	*STING1*	*COPA*
